# Absence of accessory genes in a divergent simian T-lymphotropic virus type 1 isolated from a bonnet macaque (*Macaca radiata*)

**DOI:** 10.1371/journal.pntd.0007521

**Published:** 2019-07-08

**Authors:** Philippe V. Afonso, Zahra Fagrouch, Martin Deijs, Henk Niphuis, Willy Bogers, Antoine Gessain, Lia van der Hoek, Ernst J. Verschoor

**Affiliations:** 1 Unité EPVO, Institut Pasteur, UMR 3569 CNRS, Paris, France; 2 Department of Virology, Biomedical Primate Research Centre, Rijswijk, the Netherlands; 3 Laboratory of Experimental Virology, Department of Medical Microbiology, Amsterdam UMC, University Of Amsterdam, Amsterdam, the Netherlands; WRAIR, UNITED STATES

## Abstract

**Background:**

Primate T-lymphotropic viruses type 1 (PTLV-1) are complex retroviruses infecting both human (HTLV-1) and simian (STLV-1) hosts. They share common epidemiological, clinical and molecular features. In addition to the canonical *gag*, *pol*, *env* retroviral genes, PTLV-1 purportedly encodes regulatory (i.e. Tax, Rex, and HBZ) and accessory proteins (i.e. P12/8, P13, P30). The latter have been found essential for viral persistence *in vivo*.

**Methodology/Principal findings:**

We have isolated a STLV-1 virus from a bonnet macaque (*Macaca radiata–Mra18C9*), a monkey from India. The complete sequence was obtained and phylogenetic analyses were performed. The Mra18C9 strain is highly divergent from the known PTLV-1 strains. Intriguingly, the Mra18C9 lacks the 3 accessory open reading frames. In order to determine if the absence of accessory proteins is specific to this particular strain, a comprehensive analysis of the complete PTLV-1 genomes available in Genbank was performed and found that the lack of one or many accessory ORF is common among PTLV-1.

**Conclusion:**

This study raises many questions regarding the actual nature, role and importance of accessory proteins in the PTLV-1 biology.

## Author summary

Primate T-lymphotropic viruses type 1 (PTLV-1) are complex retroviruses infecting both human (HTLV-1) and simian (STLV-1) hosts. It has been shown that the persistence and pathogenesis of these viruses depend on the expression of small, accessory proteins.

A bonnet macaque (a monkey present in India) was found infected with STLV-1. The genome was sequenced and found quite divergent from the other STLV-1 genomes previously described. Intriguingly, this virus does not encode accessory proteins. Analysis of other available sequences found that most strains lack at least one accessory gene. Thus the importance and the role of these proteins in the PTLV-1 biology should be revisited.

## Introduction

The Primate T-lymphotropic virus type 1 (PTLV-1) constitutes a group of deltaretroviruses infecting humans (HTLV-1) or non-human primates (STLV-1). Phylogenetic studies led to the definition of PTLV-1 viral subtypes [1]. Viruses belonging to the African PTLV-1 subtypes (i.e. subtypes b, d, e, f and g) are found both in humans and non-human primates (NHPs), and the human and simian strains are undistinguishable. Continuous zoonotic spillovers of these strains occur, following severe bites by infected non-human primates, or in the context of bushmeat hunting and handling [2–4]. Two subtypes are exclusively human: PTLV-1a is found in human populations scattered throughout the globe, while PTLV-1c is found in indigenous people of the Australomelanesian continent. In contrast, a group of STLV-1 has been described in macaques and great apes in Asia [5–7]. These viruses are genetically distant from other subtypes (a fact that led some researchers to consider some of them as a separate STLV, STLV-5) [8] and have never been found in humans to date.

HTLV-1 is estimated to infect at least 5–10 million people worldwide [1]. HTLV-1 is the etiological agent of many severe diseases, ranging from an aggressive lymphoproliferation, the adult T-cell leukemia/lymphoma (ATL), to inflammatory syndromes, such as a neurodegenerative disease called HTLV-1 associated myelopathy or tropical spastic paraparesis (HAM/TSP). Pathogenesis does not seem to be restricted to a certain HTLV-1 subtype; for instance, ATL cases have been reported in patients infected with HTLV-1a, -1b or 1c [9, 10]. STLV-1 is also oncogenic. ATL have been reported in many simian species, from Macaques to Gorilla [11, 12].

Deltaretroviruses are complex retroviruses. In addition to the canonical *gag*, *pol*, *env* retroviral genes, the PTLV-1 genome has a series of open reading frames (ORFs) encoding regulatory and accessory proteins [13–15]. Regulatory proteins are essential for viral expression and propagation both *in vitro* and *in vivo*. In contrast, accessory proteins are optional for viral expression *in vitro*, but required for viral persistence *in vivo*. There are three regulatory proteins in PTLV-1: Tax, Rex, and HBZ. PTLV-1 purportedly encodes four accessory proteins, named P12/P8 (encoded by ORF I), P13 and P30 (encoded by ORF II).

This study reports the first STLV-1 genome from a virus infecting a bonnet macaque (*Macaca radiata*), a macaque species from India. The virus replicated well in cell culture, and this material was used for sequencing, a full genome analysis of this divergent Asian STLV-1 strain was performed. While the canonical ORFs, as well as the ORFs encoding regulatory proteins were found conserved, the ORFs encoding the accessory proteins were absent or disrupted. This latter observation is intriguing as these proteins are purportedly essential for viral persistence *in vivo*. In order to determine if the absence of accessory proteins is specific to this particular strain, a comprehensive analysis of the complete genomes of different PTLV-1 subtypes available in GenBank was performed. Except for HTLV-1a strains, strains from other PTLV-1 subtypes all lacked at least one accessory ORF. This raises many questions regarding the actual role and importance of accessory proteins in the PTLV-1 biology.

## Results

### STLV detection in a sample from bonnet macaque

The bonnet macaque Mra18C9 was housed in an animal rescue center and material was sent to us for routine diagnostic screening.

Serological tests for PTLV-1 gave conflicting results. While the serum tested negative on an in-house ELISA, it reacted positively when tested by a local hospital laboratory. These conflicting results led us to perform additional serological testing using the INNO-LIA HTLVI/II assay (Fujirebio Europe, Ghent, Belgium). The serum reacted strongly with PTLV-1/2 env gp46 and gp21 antigens, but did not react with any type-specific peptide. It was thus considered as an indeterminate sero-reactivity.

As the serological tests were inconclusive, we performed a diagnostic PCR screening on the DNA isolated from PBMCs, by using a generic nested PCR assay able to amplify a fragment of the *tax*/*rex* region of both PTLV-I and -II. The PCR tested positive, and BLAST analysis on the 118 bp-long fragment revealed high nucleotide identity percentage (>93%) with STLV-1 identified from Formosan macaques (*Macaca cyclopis* STLV108, GenBank accession number: KM268809), stump-tailed macaques (*M*. *arctoides* MarB43 and marc1, GenBank AY590142 and U76625 respectively), and long-tailed macaques (*M*. *fascicularis* MFA-C194, GenBank U59132).

The full-length STLV-1 genome infecting the bonnet macaque Mra18C9 was obtained by using a combination of the sequence-independent VIDISCA-454 technique, which generated 4 segments scattered along the STLV genome, and standard PCRs to bridge the sequence gaps and sequence the LTR.

### Mra18C9 STLV-1 encodes the canonical retroviral proteins, as well as the regulatory proteins

The flanking long terminal repeats (LTRs) comprise a TATA box, a polyadenylation signal, and three Tax-responsive elements (TxRE) type 1. The TxRE-2 may not be fully functional as an insertion of two nucleotides is present at position 210–211. Interestingly, the LTR was quite divergent from previously published PTLV-LTR sequences, as the nucleotide identity was lower than 80% (Table A in S1 Text).

**Fig 1:**
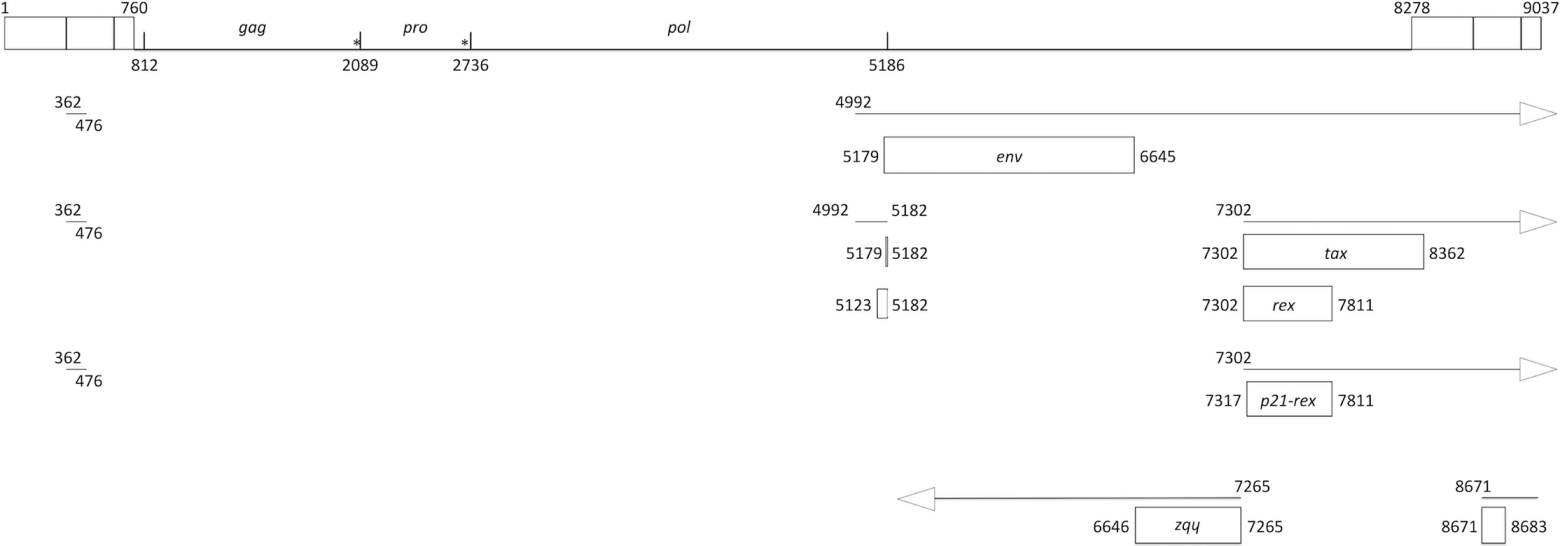
Genomic organization of STLV-1 Mra18C9. Nucleotide numbering of splicing sites and initiation/stop codon are according to the Mra18C9 proviral genome. The upper boxes represent the LTRs. The other boxes represent the ORFs. * indicate the frame-shifting sites.

The canonical retroviral ORFs (*gag*, *pro*, *pol*, *env*) are conserved, as well as the sequences necessary for the gag-pro and pro-pol ribosomal frame-shifts (Fig 1). Nucleotide and amino acid sequence comparisons confirm that Mra18C9 STLV-1 is a highly divergent PTLV-1 strain. At the nucleotide level, the *gag*, *pro*, *pol*, *and env* genes show not more than 80% identity with their counterparts from other PTLV, while their encoded proteins differ 10–15% in amino acid identity (Tables A-B in S1 Text).

The ORFs corresponding to the regulatory proteins Tax, Rex and HBZ are also present (Fig 1), and splice-acceptor and -donor sites are preserved. The *tax* gene sequence shares roughly 82% nucleotide identity, and the encoded Tax protein has a 88–90% amino-acid identity to other PTLV-1 sequences (Tables A-B in S1 Text). Importantly, the PDZ-binding motif of Tax, which is necessary for the interaction of Tax with cellular factors, is mutated in Mra18C9 genome: the ETEV motif is changed into an ETEI motif.

**Fig 2:**
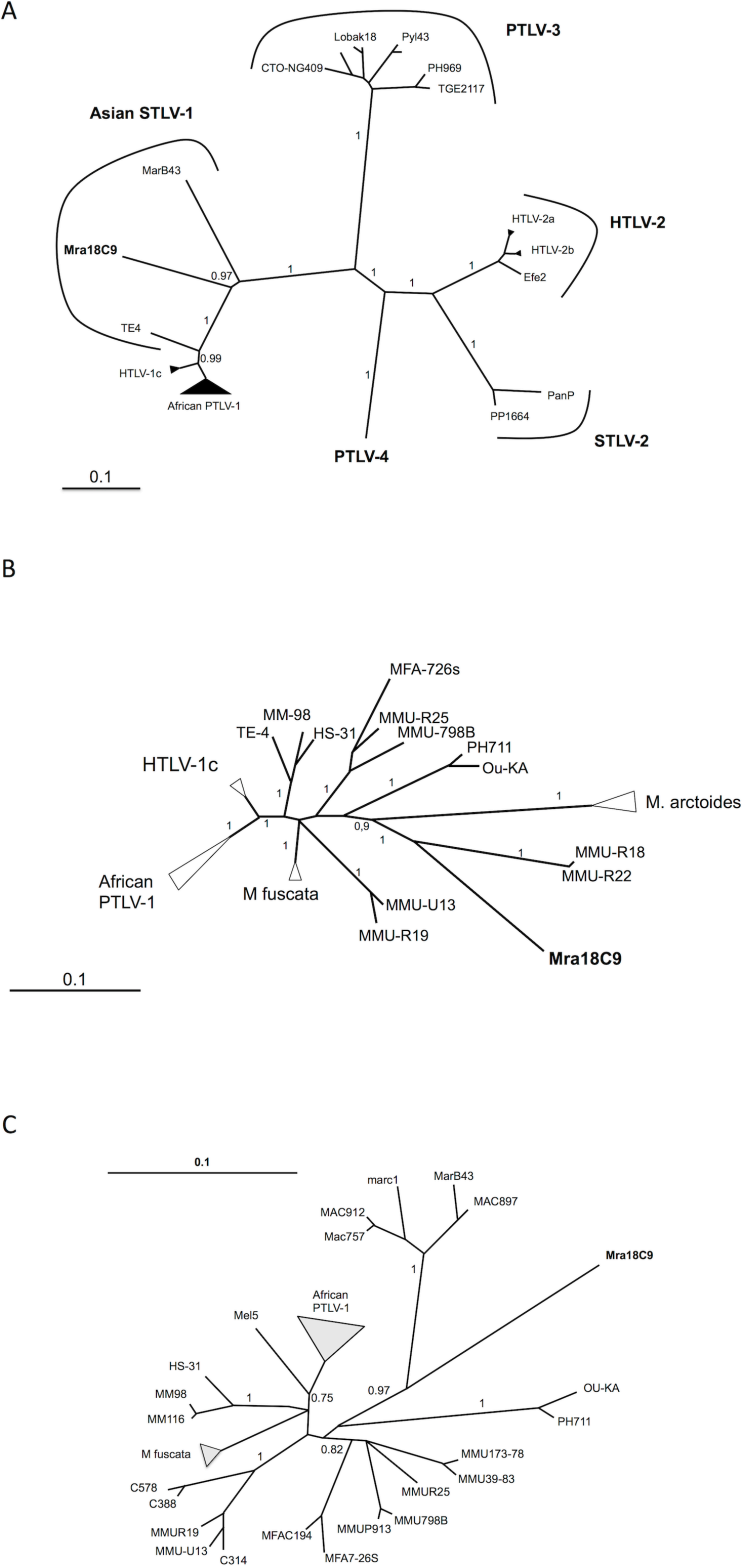
Phylogenetic analyses of the MraC18C9 STLV-1 strain. The presented phylogenetic trees were all generated by a Bayesian approach. The scale bar represents nucleotide substitutions per site. Values correspond to posterior probabilities. A—Phylogenetic tree generated by a Bayesian approach using a concatenation of *gag-pol-env-tax* genes (alignment of 5820 nucleotides). PTLV-4 sequences (GabL14, Ggo51461 and Ggo 50539) were used as outgroup. B—Phylogenetic tree generated using the LTR sequence (alignment of 701 nucleotides). The tree is unrooted and comprises most PTLV-1 complete LTR sequences available. C- Phylogenetic tree using an alignment of an *env* gene fragment of 483 nucleotides. The tree is unrooted and comprises most PTLV-1 *env* sequences available. The denomination African PTLV-1 comprises HTLV-1a, and PTLV-1b, d, e, f, and g strains. MM stands for *Macaca maura*; MMU stands for *M*. *mulatta*; MFA stands for *M*. *fascicularis*; MAC and mar stand for *Macaca arctoides*; TE stands for M. tonkeana; HS stands for *Hylobates syndactylus*; Ou stands for *Orangutan*; Mra stands for M.radiata; Cxxx strains were isolated from *Hylobates pileatus*.

Phylogenetic analyses of the concatenated *gag*-*pol*-*env*-*tax* genes clearly demonstrated that Mra18C9 falls into the Asian STLV-1 group (Fig 2A). Macaque STLV-1 strains all form long branches, and do not aggregate in a monophyletic group. Instead, they form a paraphyletic group. In order to better estimate the relative position of this strain, phylogenetic analyses were performed using the commonly studied LTR and *env* sequences (Fig 2B and 2C). The different phylogenetic methods (Neighbor-Joining, and a Bayesian approach) gave very similar results. Results of the Bayesian analyses are shown in Fig 2. The Mra18C9 strain was consistently found in a long branch among the macaque strains; the closest known sequences were the *Macaca arctoides* strains (when considering either LTR or env sequences) (Fig 2B and 2C) and the *Macaca mulata* strains MMU-R18 and R22 (when considering the LTR) (Fig 2B).

In conclusion, the Mra18C9 strain has a typical PTLV-1 organization, but this virus is highly divergent from other PTLV, suggesting a long and independent evolution.

### Mra18C9 STLV-1 lacks accessory genes

Sequence analysis suggests that the accessory proteins encoded by ORF-I may not be functional in the Mra18C9 strain. Indeed, the start codon of the open-reading frame is mutated (ATG > ^6821^GCG), and multiple stop codons are found. Furthermore, the splice-acceptor site is mutated (ATK ^6380^C**AG**/**C**AAC > Mra18C9 ^6719^T**AA**/**C**AAC) and may not be functional either. Thus, Mra18C9 does not encode the P12/P8 proteins.

The splice-acceptor domain necessary for a P30 protein-encoding mRNA is also mutated (ATK ^6475^T**AG/C**ACT > Mra18C9 ^6814^G**GA**/**C**GCT), suggesting that P30 may not be produced by the Mra18C9 strain. This is reinforced by the presence of an early stop codon in the ORF.

Similarly, the splice-acceptor domain, necessary for a P13-encoding mRNA, is also mutated (ATK ^6872^C**AG**/**C**AGG > MRA18C9 ^7223^C**AG**/**T**TGG), and therefore the mRNA may not be synthesized. In addition, the ORF is also altered: the start codon is conserved but the stop codon is mutated (TAA > ^7549^CAA). The resulting protein would then be much longer (137 amino-acid long putative protein instead of 87 aa-long), which can rigorously influence its functionality.

Collectively, our analyses indicate that the STLV-1 Mra18C9 strain completely lacks the accessory genes, which have been reported in HTLV-1a strains.

### Many published PTLV-1 strains lack one or several accessory proteins

We wondered if the absence of accessory genes was specific to the Asian macaque STLV-1 strain isolated from *M*. *radiata*. For this purpose, an *in silico* analysis of PTLV-1 complete genomes available in GenBank was performed (Table 1 and Tables C-F in S1 Text).

**Table 1:** Many PTLV-1 lack one or more accessory proteins.

	Putative protein		
	P12	P13	P30
**HTLV-1a**	present (99 aa)*	present (87 aa)	present (241 aa)
**HTLV-1b**	**absent**	present (87 aa)	**absent**
**STLV-1b**	present (86 aa)	present (87 aa)	present (241 aa)
**HTLV-1c**	**absent**	present (87 aa)	present (240 or 242 aa)
**STLV-1e**	present (99 aa)	**absent**	present (253 aa)
**STLV-1f**	**absent**	present (87 aa) or **absent**	present (241 aa)
**PTLV-1 smm**	present (86 aa)	**absent**	present (241 aa)
**STLV-1g**	present (86 aa)	**absent**	**absent**
**TE-4**	**absent**	**absent**	**absent**
**Chaplin**	**absent**	**absent**	**absent**
**MarB43**	**absent**	**absent**	**absent**

Many PTLV-1 strains (Table C in S1 Text) were analyzed and the presence of P12, P13 et P30 were addressed, focusing mostly on the conservation of the splicing sites, the presence of the start codon and the absence of early stop codon (Tables D-F in S1 Text).

* although the complete sequences of HTLV-1a all encode a 99 aa-long protein, many shorter versions of the proteins have been reported [16, 17].

Accessory proteins were conserved in HTLV-1a strains and in the available STLV-1b strain [12]. In contrast, the macaque STLV-1 strains lacked all of the accessory genes. The loss of accessory genes in MarB43 was previously reported [7]. All the other strains (either HTLV-1b, c; or STLV-1e, f, g) lacked at least 1 accessory protein. For instance, the HTLV-1b strains lack both P12 and P30, and as previously mentioned HTLV-1c strains lack P12 [18].

In conclusion, although accessory genes have been found important for viral infection and persistence in HTLV-1a, many PTLV-1 strains lack one or more accessory genes.

## Discussion

PTLV-1 infects a wide range of non-human primates (NHPs). We report the first strain infecting a bonnet macaque (*Macaca radiata*). Complete genome analysis revealed that it is a highly divergent strain when compared to the currently known PTLVs. This would explain the conflicting results obtained by ELISA, as well as its low reactivity (indeterminate profile) on the commercial Western blot. Phylogenetic analyses positioned the Mra18C9 strain among other Asian macaque STLV-1 viruses. This confirms the large heterogeneity within the Asian PTLV-1 clade as was described previously [5, 6].

In phylogenetic trees, Asian macaque STLV-1 strains form very long branches when compared to African (including HTLV-1a) strains. This could suggest that these viruses have evolved independently in Asia, with their simian host, for a very long period [19]. The introduction of STLV in Asian NHP has been dated at approximately 200.000 years ago [6, 7]. Under this assumption, the viruses have coevolved for long with their host, and the phylogeny of Asian STLV-1 should mirror the evolution of Asian primates. However, this is not the case for macaque STLV-1. First, they do not form a monophyletic group; instead they form a paraphyletic group. The strains are organized as a ladder, branching deeply next to the PTLV-1 root (Fig 2A). One could argue that this particular topology results partially from genetic saturation. Moreover, among Asian STLV-1 there are only a few monophyletic groups corresponding to simian species (Fig 2B and 2C). Apart from *M*.*fuscata* and *M*.*arctoides* STLV-1, the other clades are composed of sequences of mixed origins, with STLVs from Pongides and Hylobatides that infect macaques. Even when focusing on sequences isolated from macaques, the distribution of the sequences does not follow the known Macaca phylogeny [20, 21]. Together, this points to interspecies transmission (between macaques or from macaques to orangutans or gibbons) of such viruses, as others previously suggested [5, 6]. Interspecies transmission of STLV-1 has been previously reported, although in the context of captivity [22, 23]; the evidence of such transmission *in natura* is mostly inferred by phylogenetic analysis [6, 24]. Thus, one could argue that the long branching is not only due to a long independent evolution in Asia, but also to an accelerated mutation rate for these strains, which could be related to frequent interspecies transmission.

The canonical and the regulatory proteins are present in the genome of the Mra18C9 STLV-1. The Mra18C9 STLV-1 strain is a functional, replicative virus (at least *in vitro*, as it could be amplified on SupT1 cells). However, the strain may have an attenuated phenotype. First, the Tax-responsive element 2 (TxRE2) seems disrupted due to a 2-nucleotide insertion [25]. This may lead to a lower basal transcription and reduced viral expression [26]. Second, the viral transactivator Tax has a mutated PDZ-binding motif (PBM). The Tax PBM is essential for sustained proliferation both *in vitro* and *in vivo* [27, 28]. A single mutation of the last amino acid of the PBM was shown to be sufficient to abrogate its function [29–31].

The pX region of the Mra18C9 STLV-1 strain lacks both ORF-I and ORF-II. We first hypothesized that the loss of these ORFs could render the virus less pathogenic. Indeed, accessory proteins have been shown to be important for viral persistence and pathogenesis in HTLV-1a. Mutations of accessory ORFs limit the replicative capacity of HTLV-1a in a rabbit model [32]. Similarly, in the closely related bovine leukemia virus, mutations of the homologue ORFs render the virus attenuated [33]. It was proposed that Australian HTLV-1c strains, because they lack the P12 protein, might be less oncogenic [18]. Nevertheless, ATL cases have been reported in HTLV-1c-infected individuals [9]. Moreover, while the absence of accessory proteins seems to be a general feature of macaque STLV-1, ATL cases have been reported in naturally infected macaques [11]. Thus, even in the absence of accessory proteins, STLV-1 still present an oncogenic potential.

Although STLV-1 is highly prevalent among Asian NHPs, and humans are in frequent contact with macaques and may acquire other retroviral infection, such as simian foamy viruses that are endemic in several species of Asians monkeys [34], no Asian STLV zoonotic transmission has been reported so far. One could hypothesize that the absence of ORFI and ORFII in macaque STLV-1 can limit viral transmission and propagation. Indeed, HTLV-1a P12 and P30 have been found to be essential for viral replication in human and macaque dendritic cells [35], which can play a key role in viral transmission. However, this proposition is nullified by our thorough analysis of the different PTLV-1 subtypes. Indeed, HTLV-1b, which is a very common virus in Central Africa, persists and propagates in humans despite the absence of P12 and P30 (Table 1).

In conclusion, the role and importance of accessory proteins needs to be reconsidered in light of the analysis of the different PTLV-1 strains. Indeed, as most studies have focused on HTLV-1a, the 3 accessory proteins were found conserved and their function in viral persistence and transmission was believed to be essential [17, 32, 35]. This study indicates that some PTLV-1 can persist in the absence of one or many accessory proteins. This raises the question of a dispensable role of these proteins, or the presence of other accessory proteins yet to be identified in the other PTLV-1 subtypes.

## Methods

### Ethics statement

The Bonnet macaque was housed in an animal rescue center (animal shelter VZW, Belgium) and material was sent to the BPRC for viral diagnostic screening.

The BPRC is fully licensed by the Netherlands Food and Consumer Product Safety Authority (belonging to the Ministry of Agriculture, Nature and Food Quality) to work with animal products and perform diagnostic services for third parties (Approval no. 1926950).

### HTLV diagnosis

Sera were assayed for antibodies to PTLV-1 using an in-house developed serological test with purified, lysed HTLV-1 particles as coating antigen (Advanced Biotechnologies Inc., Eldersburg, USA) [36]. Additionally, the serum from the bonnet macaque was also tested by ELISA in a local hospital laboratory (DDL, Delft, The Netherlands), and a western blot (WB) analysis using the INNO-LIA HTLVI/II assay (Fujirebio Europe, Gent, Belgium).

Genomic DNA was isolated from whole blood using the QIAamp DNA blood mini kit (QIAGEN Benelux B.V., Venlo, The Netherlands). A 118 bp tax/rex gene fragment was amplified using the TR101/TR102 and SK43/SK44 nested primer sets, as previously described [37, 38]. Furthermore, A pan-STLV PCR was performed with primers PH1F and PH2R, as described by van Dooren *et a*l. [39]. The amplified fragment of 192 bp fully overlapped the 118 bp fragment from the first PCR.

### Virus isolation and sequencing

The virus discovery method VIDISCA-454 was used for the analysis of cell culture supernatant from the PTLV-infected SupT1 cell culture, as previously described [40]. In brief, PBMCs were isolated on a Ficoll and stimulated for 2 days with PHA (1 ug/ml final). Next, they were co-cultured with SupT1 cells until CPE was visible (2–3 weeks). The cell culture supernatant was centrifuged to remove cell debris and treated with TURBO DNase (Ambion, Thermo Fisher Scientific, Breda, The Netherlands). Next, nucleic acids were isolated with a QIAamp Viral RNA Mini Kit (QIAGEN Benelux BV, Venlo, the Netherlands) and reverse-transcribed with SuperScript II (Thermo Fisher Scientific) using non-ribosomal random hexamers. Subsequently, second strand DNA synthesis was performed with 5 U of Klenow fragment (New England Biolabs, Bioke, Leiden, The Netherlands). Double-stranded DNA was purified by phenol/chloroform extraction and ethanol precipitation and digested with Mse I restriction enzyme (New England Biolabs). Adaptors with different Multiplex Identifier sequences (MIDs) were ligated to the digested fragments of the different samples. Before PCR amplification, the fragments were purified with AMPure XP beads (Agencourt AMPure XP PCR, Beckman Coulter, Woerden, The Netherlands).

A 28-cycles PCR with adaptor-annealing primers was performed. The program of the PCR-reaction was: 5 min 95°C, and cycles of 1 min 95°C, 1 min 55°C, and 2 min 72°C, followed by 10 min 72°C and 10 min 4°C. After purification with AMPure XP beads, the purified DNA was quantified with the Quant-it dsDNA HS Qubit kit (Invitrogen, Carlsbad, CA, USA) and diluted to 10^7^ copies/μl. Samples were pooled and Kapa PCR (Kapa Biosystems, Wilmington, MA, USA) was performed to determine the quantity of amplifiable DNA in each pool. Subsequently, the Bioanalyser (hsDNA chip, Agencourt) was used to determine the average nucleotide length of the libraries. The pools were diluted until 10^6^ copies/μl, titrated with beads (DNA:beads ratio of 0.5:1, 1:1, 2:1 and 4:1) and used in an emulsion PCR according to the supplier’s protocol (LIB-A SV emPCR kit). Sequencing was done on a 2 region GS FLX Titanium PicoTiterPlate (70x75) with the GS FLX Titanium XLR 70 Sequencing kit (Roche, Woerden, The Netherlands). Sequence reads were analyzed using the blastn and blastp algorithms (National Center for Biotechnology Information).

PCR primers were designed on basis of the four fragments that were obtained from the VIDISCA as well as from the consensus LTR sequence derived from the alignment of other PTLV-1 (Tables G-H in S1 Text). PCR reactions were performed in a final volume of 50 μl. Each amplification reaction was performed in 1x DreamTaq buffer containing 200 μM of each dNTP, 50 pmol of each primer, and 1.25 U DreamTaq DNA polymerase (Thermo Fisher Scientific). The amplification reactions were performed for 35 cycles consisting of a 30 s denaturation step at 94°C, a 30 s annealing step at 55°C and an elongation step of 150 sec at 72°C. Amplicon purification and sequencing was performed essentially as described above, but by using a primer-walking sequencing strategy. Sequences were assembled with the SeqMan Pro software (DNASTAR, Inc.,Madison, USA). The resulting contig was further analyzed using the MacVector software package (MacVector, Inc., Cambridge, UK). The complete genome of STLV-1 Mra18C9 has been deposited at GenBank with accession number MK639100.

### Phylogenetic analyses

LTR (701 bases) and env (483 bases) sequences were aligned together with most sequences available on GenBank using DAMBE [41]. An alignment of concatenated gag-pol-env-tax sequences (5820 bases) was also generated with the sequences of complete PTLV-1 genomes available on GenBank.

Phylogenetic trees resulted from analyses using the neighbor-joining method performed with the PAUP* v4.0b10. The final alignment was submitted to the Modeltest program (version 3.6) to select, according to the Akaike information criterion, the best model to apply to phylogenetic analyses. The selected substitution models were: Tamura-Nei for *env* and concatenated *gag-pol-env-tax*, and general time-reversible (GTR + γ) for the LTR. To test the robustness of the tree topologies, 1,000 bootstrap replicates were performed. Bayesian approaches were inferred with the MrBayes 3.2.7 program and robustness was tested with posterior probabilities. Both methods raised similar phylogenetic tree topology.

### Analysis of ORF in PTLV-1 complete genomes

The analysis was performed following the alignment of complete genomes available in GenBank (Table C in S1 Text). Splicing acceptor and donor sequences were previously described for ATK [13]. Frameshift sites had been previously identified [42].

## Supporting information

S1 Text Supplementary tables. (PDF)

## References

[1] Gessain A, Cassar O. Epidemiological Aspects and World Distribution of HTLV-1 Infection. Front Microbiol. 2012;3:388 10.3389/fmicb.2012.00388 23162541 PMC3498738

[2] Filippone C, Betsem E, Tortevoye P, Cassar O, Bassot S, Froment A, et al A Severe Bite From a Nonhuman Primate Is a Major Risk Factor for HTLV-1 Infection in Hunters From Central Africa. Clin Infect Dis. 2015;60(11):1667–76. 10.1093/cid/civ145 . 25722199

[3] Mossoun A, Calvignac-Spencer S, Anoh AE, Pauly MS, Driscoll DA, Michel AO, et al Bushmeat Hunting and Zoonotic Transmission of Simian T-Lymphotropic Virus 1 in Tropical West and Central Africa. J Virol. 2017;91(10). 10.1128/JVI.02479-16 28298599 PMC5411610

[4] Wolfe ND, Heneine W, Carr JK, Garcia AD, Shanmugam V, Tamoufe U, et al Emergence of unique primate T-lymphotropic viruses among central African bushmeat hunters. Proc Natl Acad Sci U S A. 2005;102(22):7994–9. 10.1073/pnas.0501734102 15911757 PMC1142377

[5] Reid MJ, Switzer WM, Schillaci MA, Ragonnet-Cronin M, Joanisse I, Caminiti K, et al Detailed phylogenetic analysis of primate T-lymphotropic virus type 1 (PTLV-1) sequences from orangutans (Pongo pygmaeus) reveals new insights into the evolutionary history of PTLV-1 in Asia. Infect Genet Evol. 2016;43:434–50. 10.1016/j.meegid.2016.05.036 . 27245152 PMC11332081

[6] Van Dooren S, Verschoor EJ, Fagrouch Z, Vandamme AM. Phylogeny of primate T lymphotropic virus type 1 (PTLV-1) including various new Asian and African non-human primate strains. Infect Genet Evol. 2007;7(3):374–81. 10.1016/j.meegid.2006.06.003 . 16931175

[7] Van Dooren S, Meertens L, Lemey P, Gessain A, Vandamme AM. Full-genome analysis of a highly divergent simian T-cell lymphotropic virus type 1 strain in Macaca arctoides. J Gen Virol. 2005;86(Pt 7):1953–9. 10.1099/vir.0.80520-0 . 15958673

[8] Liegeois F, Lafay B, Switzer WM, Locatelli S, Mpoudi-Ngole E, Loul S, et al Identification and molecular characterization of new STLV-1 and STLV-3 strains in wild-caught nonhuman primates in Cameroon. Virology. 2008;371(2):405–17. 10.1016/j.virol.2007.09.037 . 17976676

[9] Einsiedel L, Cassar O, Bardy P, Kearney D, Gessain A. Variant human T-cell lymphotropic virus type 1c and adult T-cell leukemia, Australia. Emerg Infect Dis. 2013;19(10):1639–41. 10.3201/eid1910.130105 24047544 PMC3810736

[10] Mahieux R, Ibrahim F, Mauclere P, Herve V, Michel P, Tekaia F, et al Molecular epidemiology of 58 new African human T-cell leukemia virus type 1 (HTLV-1) strains: identification of a new and distinct HTLV-1 molecular subtype in Central Africa and in Pygmies. J Virol. 1997;71(2):1317–33. 8995656 10.1128/jvi.71.2.1317-1333.1997 PMC191187

[11] Homma T, Kanki PJ, King NW Jr., Hunt RD, O'Connell MJ, Letvin NL, et al Lymphoma in macaques: association with virus of human T lymphotrophic family. Science. 1984;225(4663):716–8. 10.1126/science.6087453 . 6087453

[12] Ayouba A, Michem A, Peeters M, Vercammen F. Full-Genome Characterization of Simian T-Cell Leukemia Virus Type 1 Subtype b from a Wild-Born Captive Gorilla gorilla gorilla with T-Cell Lymphoma. Genome Announc. 2017;5(43). 10.1128/genomeA.01117-17 29074651 PMC5658489

[13] Ciminale V, Pavlakis GN, Derse D, Cunningham CP, Felber BK. Complex splicing in the human T-cell leukemia virus (HTLV) family of retroviruses: novel mRNAs and proteins produced by HTLV type I. J Virol. 1992;66(3):1737–45. 1310774 10.1128/jvi.66.3.1737-1745.1992 PMC240923

[14] Koralnik IJ, Gessain A, Klotman ME, Lo Monico A, Berneman ZN, Franchini G. Protein isoforms encoded by the pX region of human T-cell leukemia/lymphotropic virus type I. Proc Natl Acad Sci U S A. 1992;89(18):8813–7. 10.1073/pnas.89.18.8813 1528897 PMC50011

[15] Gaudray G, Gachon F, Basbous J, Biard-Piechaczyk M, Devaux C, Mesnard JM. The complementary strand of the human T-cell leukemia virus type 1 RNA genome encodes a bZIP transcription factor that down-regulates viral transcription. J Virol. 2002;76(24):12813–22. 10.1128/JVI.76.24.12813-12822.2002 12438606 PMC136662

[16] Barreto FK, Khouri R, Rego FFA, Santos LA, Castro-Amarante MF, Bialuk I, et al Analyses of HTLV-1 sequences suggest interaction between ORF-I mutations and HAM/TSP outcome. Infect Genet Evol. 2016;45:420–5. 10.1016/j.meegid.2016.08.020 27553711 PMC5123959

[17] Rosadas C, Vicente ACP, Zanella L, Cabral-Castro MJ, Peralta JM, Puccioni-Sohler M. First report of HTLV-1 truncated p12 protein in Brazil. Virulence. 2017;8(7):1445–9. 10.1080/21505594.2016.1267895 27960650 PMC5711409

[18] Einsiedel L, Purcell D, Schinke S, Haynes K, Taylor GP, Martin F. Highlights from the HTLV-1 symposium at the 2017 Australasian HIV and AIDS Conference held jointly with the 2017 Australasian Sexual Health Conference, November 2017, Canberra, Australia. J Virus Erad. 2018;4(1):48–50. 29568554 10.1016/S2055-6640(20)30242-9 PMC5851185

[19] Song KJ, Nerurkar VR, Saitou N, Lazo A, Blakeslee JR, Miyoshi I, et al Genetic analysis and molecular phylogeny of simian T-cell lymphotropic virus type I: evidence for independent virus evolution in Asia and Africa. Virology. 1994;199(1):56–66. 10.1006/viro.1994.1097 . 8116255

[20] Li J, Han K, Xing J, Kim HS, Rogers J, Ryder OA, et al Phylogeny of the macaques (Cercopithecidae: Macaca) based on Alu elements. Gene. 2009;448(2):242–9. 10.1016/j.gene.2009.05.013 19497354 PMC2783879

[21] Li QQ, Zhang YP. Phylogenetic relationships of the macaques (Cercopithecidae: Macaca), inferred from mitochondrial DNA sequences. Biochem Genet. 2005;43(7–8):375–86. 10.1007/s10528-005-6777-z . 16187162

[22] Voevodin A, Samilchuk E, Schatzl H, Boeri E, Franchini G. Interspecies transmission of macaque simian T-cell leukemia/lymphoma virus type 1 in baboons resulted in an outbreak of malignant lymphoma. J Virol. 1996;70(3):1633–9. 8627684 10.1128/jvi.70.3.1633-1639.1996 PMC189987

[23] Parrish SW, Brown AE, Chanbancherd P, Gettayacamin M, Parrish JH. Transmission of STLV in a closed colony of macaques. Am J Primatol. 2004;63(2):103–9. 10.1002/ajp.20043 . 15195332

[24] Koralnik IJ, Boeri E, Saxinger WC, Monico AL, Fullen J, Gessain A, et al Phylogenetic associations of human and simian T-cell leukemia/lymphotropic virus type I strains: evidence for interspecies transmission. J Virol. 1994;68(4):2693–707. 7908063 10.1128/jvi.68.4.2693-2707.1994 PMC236747

[25] Fujisawa J, Toita M, Yoshida M. A unique enhancer element for the trans activator (p40tax) of human T-cell leukemia virus type I that is distinct from cyclic AMP- and 12-O-tetradecanoylphorbol-13-acetate-responsive elements. J Virol. 1989;63(8):3234–9. 2545901 10.1128/jvi.63.8.3234-3239.1989 PMC250893

[26] Tanimura A, Teshima H, Fujisawa J, Yoshida M. A new regulatory element that augments the Tax-dependent enhancer of human T-cell leukemia virus type 1 and cloning of cDNAs encoding its binding proteins. J Virol. 1993;67(9):5375–82. 8350401 10.1128/jvi.67.9.5375-5382.1993 PMC237938

[27] Xie L, Yamamoto B, Haoudi A, Semmes OJ, Green PL. PDZ binding motif of HTLV-1 Tax promotes virus-mediated T-cell proliferation in vitro and persistence in vivo. Blood. 2006;107(5):1980–8. 10.1182/blood-2005-03-1333 16263794 PMC1895710

[28] Peres E, Blin J, Ricci EP, Artesi M, Hahaut V, Van den Broeke A, et al PDZ domain-binding motif of Tax sustains T-cell proliferation in HTLV-1-infected humanized mice. PLoS Pathog. 2018;14(3):e1006933 10.1371/journal.ppat.1006933 29566098 PMC5882172

[29] Rousset R, Fabre S, Desbois C, Bantignies F, Jalinot P. The C-terminus of the HTLV-1 Tax oncoprotein mediates interaction with the PDZ domain of cellular proteins. Oncogene. 1998;16(5):643–54. 10.1038/sj.onc.1201567 . 9482110

[30] Hirata A, Higuchi M, Niinuma A, Ohashi M, Fukushi M, Oie M, et al PDZ domain-binding motif of human T-cell leukemia virus type 1 Tax oncoprotein augments the transforming activity in a rat fibroblast cell line. Virology. 2004;318(1):327–36. 10.1016/j.virol.2003.10.006 . 14972558

[31] Tsubata C, Higuchi M, Takahashi M, Oie M, Tanaka Y, Gejyo F, et al PDZ domain-binding motif of human T-cell leukemia virus type 1 Tax oncoprotein is essential for the interleukin 2 independent growth induction of a T-cell line. Retrovirology. 2005;2:46 10.1186/1742-4690-2-46 16042787 PMC1199618

[32] Lairmore MD, Albrecht B, D'Souza C, Nisbet JW, Ding W, Bartoe JT, et al In vitro and in vivo functional analysis of human T cell lymphotropic virus type 1 pX open reading frames I and II. AIDS Res Hum Retroviruses. 2000;16(16):1757–64. 10.1089/08892220050193272 . 11080823

[33] Willems L, Kerkhofs P, Dequiedt F, Portetelle D, Mammerickx M, Burny A, et al Attenuation of bovine leukemia virus by deletion of R3 and G4 open reading frames. Proc Natl Acad Sci U S A. 1994;91(24):11532–6. 10.1073/pnas.91.24.11532 7972096 PMC45265

[34] Jones-Engel L, Engel GA, Schillaci MA, Rompis A, Putra A, Suaryana KG, et al Primate-to-human retroviral transmission in Asia. Emerg Infect Dis. 2005;11(7):1028–35. 10.3201/eid1107.040957 16022776 PMC3371821

[35] Valeri VW, Hryniewicz A, Andresen V, Jones K, Fenizia C, Bialuk I, et al Requirement of the human T-cell leukemia virus p12 and p30 products for infectivity of human dendritic cells and macaques but not rabbits. Blood. 2010;116(19):3809–17. 10.1182/blood-2010-05-284141 20647569 PMC2981536

[36] Warren KS, Niphuis H, Heriyanto, Verschoor EJ, Swan RA, Heeney JL. Seroprevalence of specific viral infections in confiscated orangutans (Pongo pygmaeus). J Med Primatol. 1998;27(1):33–7. . 9606041 10.1111/j.1600-0684.1998.tb00066.x

[37] Maloney EM, Biggar RJ, Neel JV, Taylor ME, Hahn BH, Shaw GM, et al Endemic human T cell lymphotropic virus type II infection among isolated Brazilian Amerindians. J Infect Dis. 1992;166(1):100–7. 10.1093/infdis/166.1.100 . 1607683

[38] Giri A, Slattery JP, Heneine W, Gessain A, Rivadeneira E, Desrosiers RC, et al The tax gene sequences form two divergent monophyletic lineages corresponding to types I and II of simian and human T-cell leukemia/lymphotropic viruses. Virology. 1997;231(1):96–104. 10.1006/viro.1997.8511 . 9143307

[39] Van Dooren S, Shanmugam V, Bhullar V, Parekh B, Vandamme AM, Heneine W, et al Identification in gelada baboons (Theropithecus gelada) of a distinct simian T-cell lymphotropic virus type 3 with a broad range of Western blot reactivity. J Gen Virol. 2004;85(Pt 2):507–19. 10.1099/vir.0.19630-0 . 14769908

[40] de Vries M, Deijs M, Canuti M, van Schaik BD, Faria NR, van de Garde MD, et al A sensitive assay for virus discovery in respiratory clinical samples. PLoS One. 2011;6(1):e16118 10.1371/journal.pone.0016118 21283679 PMC3025933

[41] Xia X, Xie Z. DAMBE: software package for data analysis in molecular biology and evolution. J Hered. 2001;92(4):371–3. 10.1093/jhered/92.4.371 . 11535656

[42] Nam SH, Kidokoro M, Shida H, Hatanaka M. Processing of gag precursor polyprotein of human T-cell leukemia virus type I by virus-encoded protease. J Virol. 1988;62(10):3718–28. 2843670 10.1128/jvi.62.10.3718-3728.1988 PMC253515

