# Supplementary material for: Absence of accessory genes in a divergent simian T-lymphotropic virus type 1 isolated from a bonnet macaque (Macaca radiata)
Source: PLoS Negl Trop Dis. 2019 Jul 8;13(7):e0007521. doi: 10.1371/journal.pntd.0007521 (PMC6638983; doi:10.1371/journal.pntd.0007521)
Supplement: S1 Text — (PDF) [file pntd.0007521.s001.pdf]

S1 Text

Absence of accessory genes in a divergent simian T-lymphotropic virus type 1 isolated from a bonnet macaque (*Macaca radiata*).

Philippe V. Afonso, Zahra Fagrouch, Martin Deijs, Henk Niphuis, Willy Bogers, Antoine Gessain, Lia van der Hoek, Ernst J. Verschoor

	LTR	gag	pol	*env*	*tax*
**ATK**	77,6	78,8	76,3	78,3	82,9
**Mel5**	79,32	79,4	76,2	78,5	83,2
**TE4**	77,86	80	76,8	78,1	82,4
**MarB43**	72,1	76,63	74,9	78,3	81,5

Table A – Percentage of nucleotide identity between Mra18C9 STLV-1 genes and 4 reference strains.
ATK is the reference HTLV-1a strain; Mel5 is a HTLV-1c strain; MarB43 and TE4 are the 2 Macaque STLV-1 complete genomes available in Genbank.

	GAG	POL	ENV	TAX
**ATK**	87,9	81,5	85,8	88,1
**Mel5**	88,8	82,6	87,2	89,3
**TE4**	89	81,26	86	88,7
**MarB43**	85,4	80	86	87,6

Table B – Percentage of amino acid (B) identity between Mra18C9 STLV-1 genes and 4 reference strains.
ATK is the reference HTLV-1a strain; Mel5 is a HTLV-1c strain; MarB43 and TE4 are the 2 Macaque STLV-1 complete genomes available in Genbank.

subtype	Name	Genbank acc. Nb	host	country
HTLV-1a	ATK	J02029	human	Japan
	WHP	AF259264		China
	BOI	L36905		Caribbean
	RKI3-Ger	AF042071		Germany (Romanian patient)
	HTVPRCAR	NC001436		Caribbean
	Waziri	AF139170		USA
	ASY032	KF797837		Brazil
	ASY042	KF797841		Brazil
	ATL005	KF797887		Brazil
	ATL003	KF797859		Brazil
	1066/05	HQ606137		Canada
	1067/05	HQ606138		Canada
	CV79	KX430031		Cape Verde
HTLV-1b	SF26	JX507077		Brazil
	EL	M67514		DRC
HTLV-1c	mel5	L02534		Solomon Island
	Aus-DF	KF242505		Australia
	Aus-CS	KF242506		Australia
	Aus-NR	JX891479		Australia
	Aus-GM	JX891478		Australia
	Van-EM5	KX905202		Australia
	NCP201	KX905203		New Caledonia
PTLV-1smm	PTLV-1smm	KU214243		USA (patient from Liberia)
STLV-1b	M10431	MF622054	Gorilla Gorilla	Cameroon (wild born)
STLV-1e	Baboon_12752	MF621979	Papio anubis	USA (captivity)
STLV-1f	Baboon_F88395	JX987040		
	Baboon_12141	MF621980		
STLV-1g	Tan90	NC000858	Cercopithecus tantalus	CAR (wild)
STLV-1mac	TE4	Z46900	Macaca tonkeana	France (captivity)
	**Mra18C9**	**MK639100**	**Macaca radiata**	Belgium (animal shelter)
	MarB43	AY590142	Macaca arctoides	France (captivity)

Table C– List of the complete genomes analyzed in the study.
The subtype, name, and accession number of the different strains are presented. The origin of the sample (host, country of collection) are also displayed.

	**splicing acceptor** (position 6383)	**start codon (ATG)**	**putative protein**
**HTLV-1a**	yes	yes	present (99 aa)*
**HTLV-1b**	yes	no	**absent**
**SLTV-1b**	yes	yes	present (86 aa)
**HTLV-1c**	yes	no	**absent**
**STLV-1e**	yes	yes	present (99 aa)
**STLV-1f**	yes	no	**absent**
**PTLV-1 smm**	yes	yes	present (86 aa)
**STLV-1g**	yes	yes	present (86 aa)
**TE-4**	yes	no	**absent**
**Mra18C9**	mutated	-	**absent**
**MarB43**	mutated	-	**absent**

Table D- Many PTLV-1 lack P12.

Many PTLV-1 strains were analyzed and the presence of P12 was addressed. We focused on the conservation of the splicing acceptor (position 6383 on the ATK reference strain), and the presence of the start codon. * although the complete sequences of HTLV-1a all encode a 99 aa-long protein, many shorter versions of the proteins have been reported [10, 11].

	**splicing acceptor** (position 6478)	**start codon (ATG)**	**putative protein**
**HTLV-1a**	yes	yes	present (241 aa)
**HTLV-1b**	mutated	-	**absent**
**SLTV-1b**	yes	yes	present (241 aa)
**HTLV-1c**	yes	yes	present (240 or 242 aa)
**STLV-1e**	yes	yes	present (253 aa)
**STLV-1f**	yes	yes	present (241 aa)
**PTLV-1 smm**	yes	yes	present (241 aa)
**STLV-1g**	yes	no	**absent**
**TE-4**	yes	yes	**absent (multiple early stop codon)**
**Chaplin**	mutated	-	**absent**
**MarB43**	mutated	-	**absent**

Table E- Many PTLV-1 lack P30.

Many PTLV-1 strains were analyzed and the presence of P30 was addressed. We focused on the conservation of the splicing acceptor (position 6478 on the ATK reference strain), and the presence of the start codon.

	**splicing acceptor** (position 6875)	**start codon (ATG)**	**putative protein**
**HTLV-1a**	yes	yes	present (87 aa)
**HTLV-1b**	yes	yes	present (87 aa)
**SLTV-1b**	yes	yes	present (87 aa)
**HTLV-1c**	yes	yes	present (87 aa)
**STLV-1e**	yes	no	**absent**
**STLV-1f**	yes	1 out of 2	present (87 aa) or **absent**
**PTLV-1 smm**	yes	no	**absent**
**STLV-1g**	yes	no	**absent**
**TE-4**	yes	no	**absent**
**Mra18C9**	mutated	-	**absent**
**MarB43**	mutated	-	**absent**

Table F- Many PTLV-1 lack P13.

Many PTLV-1 strains were analyzed and the presence of P13 was addressed. We focused on the conservation of the splicing acceptor (position 6875 on the ATK reference strain), and the presence of the start codon.

	Relative to STLV-1 MarB43 genome
VIDISCA-1	2389-2886
VIDISCA-2	4694-4912
VIDISCA-3	6309-6511
panSTLV	7379-7571
VIDISCA-4	8062-8325

Table G- Locations of the sequences obtained by VIDISCA and the diagnosis PCR.

These sequences, together with the consensus LTR sequence derived from the alignment of other PTLV-1 were used to design the primers in S7 Table.

Fragment	Forward primer	Position*	Reverse primer	Position*	Expected size (bp)*
1	ACTTCCTCCCCATGTTTGTC	223-242	AGGTGTTCTAGCCCTAACAG	2584-2603	2380
2	CTGTTGTTCTCACATCTTGCC	2441-2461	TGAGCTAGTTGGGTTGTACG	4780-4799	2358
3	GCCCCGCCTACGTTTCCCAG	4708-4727	GGATGGAAACATGAGAGTTG	6441-6460	1752
4	AATTGCCCAGTATGCTGCCC	6333-6352	AAGGCGTGACGATGTAGGCG	7477-7496	1163
5	GACAGAGCCTCCTTTACG	7385-7402	GACCGTGGGAGAGCAGGAACG	8228-8248	863
6	TCACTGATGGCACGCCCATG	8111-8130	GGATGGCGGCCTCAGGTAGG	8726-8745	634

Table H – PCR primer pairs used for amplification of overlapping sub-genomic STLV fragments. * Nucleotide numbering based on STLV-1 MarB43 genome (acc. no. AY590142)
